# First person – Samantha Payne

**DOI:** 10.1242/bio.050302

**Published:** 2020-01-14

## Abstract

First Person is a series of interviews with the first authors of a selection of papers published in Biology Open, helping early-career researchers promote themselves alongside their papers. Samantha Payne is first author on ‘Defined extracellular ionic solutions to study and manipulate the cellular resting membrane potential’, published in BiO. Samantha is a postdoctoral scholar in the lab of Madeleine Oudin at Tufts University, Medford, USA, investigating bioelectric signaling in cancer and regeneration.


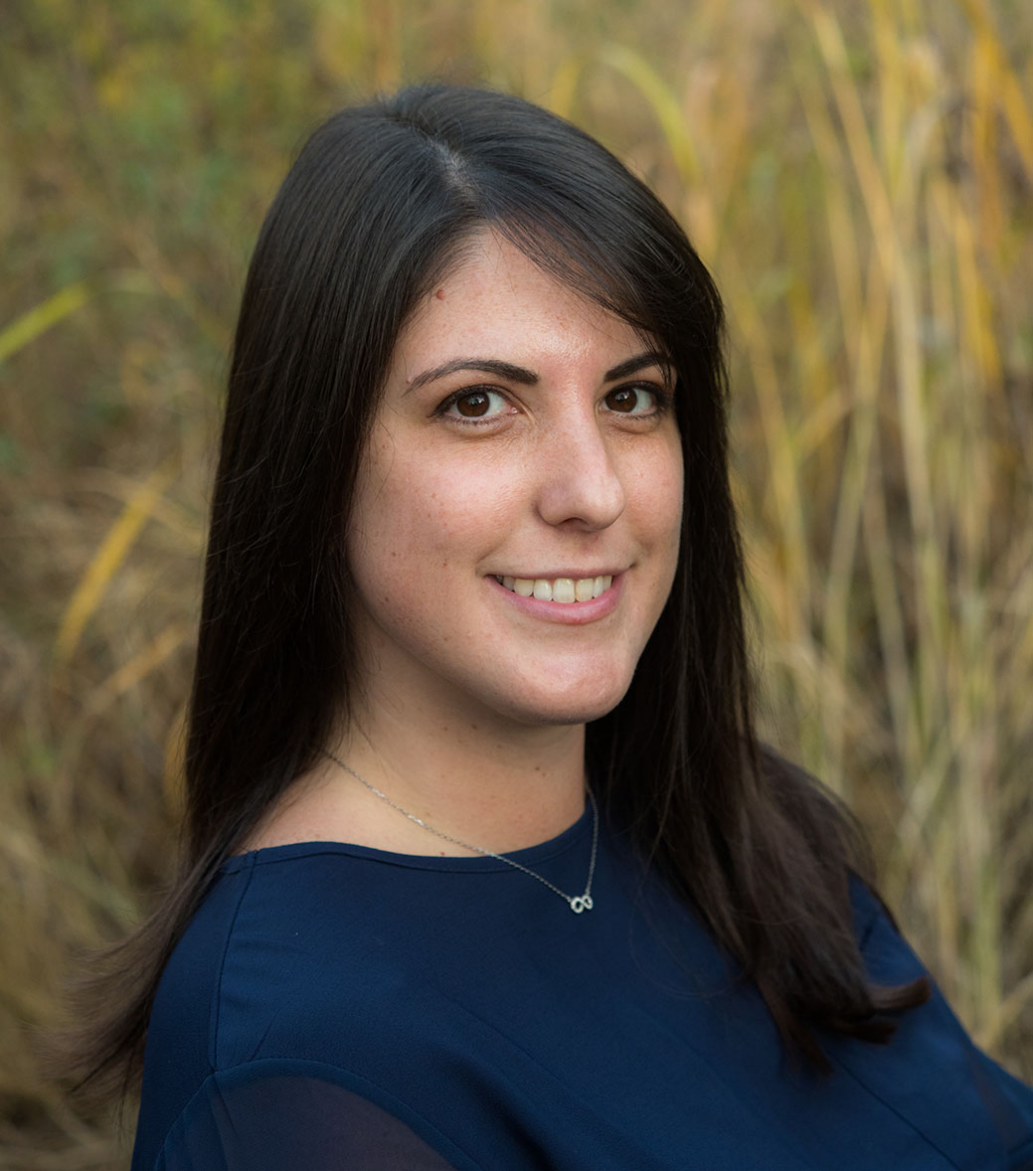


**Samantha Payne**

**What is your scientific background and the general focus of your lab?**

I'm currently studying bioelectric signaling in breast cancer metastasis, and how we might control and prevent the spread of breast cancer by targeting bioelectric-driven pathways of cell migration. The Oudin lab focuses on how metastasis can be controlled by different components of the tumor microenvironment – from extracellular matrix to stromal cell interactions. My scientific background includes the study of regeneration in non-mammalian species, as well as stem cell delivery to treat stroke. Although these may seem like very different areas of research, there are many common molecular pathways shared by cancer and stem cells and exploiting these pathways may lead to new therapies for both cancer and regeneration.

**How would you explain the main findings of your paper to non-scientific family and friends?**

Ion channels are proteins that control the flow of different ions in and out of a cell. Ions are important for many cellular processes, such as proliferation and migration. Our paper introduces a simple technique to measure the activity of ion channels of any cell type. We demonstrate that with this method we can identify which type of ion channel is important in controlling the electrical activity of the cell, and thus downstream signaling pathways. Understanding which ion channels are important can lead to the development of new targets in a wide number of disease states.

**What are the potential implications of these results for your field of research?**

It is becoming increasingly clear that ion channels and bioelectric signals are important in many cellular processes and are often altered in disease states. However, current techniques for determining ion channel activity are low-throughput and technically challenging. Our proposed method increases the accessibility of this technique to allow more research to be conducted in this exciting emerging field.

**Figure:**
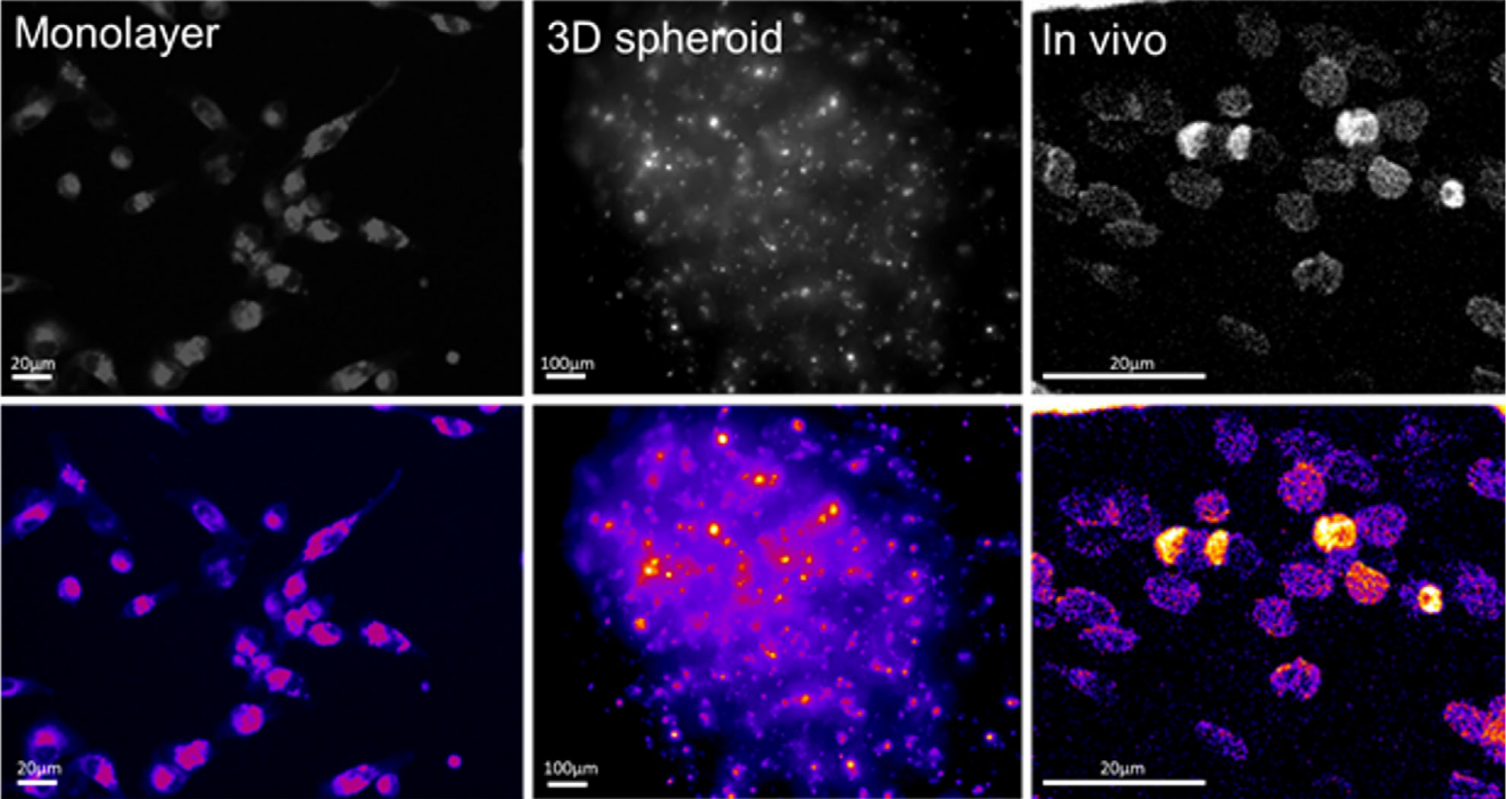
**A voltage-sensitive dye can be used to visualize the bioelectric properties of human breast cancer cells in 2D monolayer (left), 3D spheroid (middle) and *in vivo* (right).** A false-color filter has been applied to illustrate the differences in bioelectric signal between cells.

**What's next for you?**

I hope to one day start my own academic lab pursuing the common mechanisms between cancer and regeneration and how we may exploit them for therapeutic purposes.
